# Supplementary material for: An Interpretable Radiomics Model Based on Two-Dimensional Shear Wave Elastography for Predicting Symptomatic Post-Hepatectomy Liver Failure in Patients with Hepatocellular Carcinoma
Source: Cancers (Basel). 2023 Nov 6;15(21):5303. doi: 10.3390/cancers15215303 (PMC10647503; doi:10.3390/cancers15215303)
Supplement: Supplementary file 1 [file cancers-15-05303-s001.zip › cancers-2654149-supplementary.pdf]

An Interpretable Radiomics Model Based on Two-dimensional Shear Wave Elastography for Predicting Symptomatic Post-hepatectomy Liver Failure in Patients with Hepatocellular Carcinoma

Supplementary Material

Method S1. Formulas for Child-Pugh score, ALBI score and MELD score

(1) The Child-Pugh score was based on the total bilirubin, albumin, prothrombin time, and the clinical findings of encephalopathy and ascites. It was graded as 5–6 points for Child-Pugh grade A; 7–9 points for Child-Pugh grade B; and 10–15 points for Child-Pugh grade C.

(2) The following formula determined the ALBI score: (log10 bilirubin μmol/L × 0.66) + (−0.085 × albumin g/L). The ALBI score was graded as: score ≤−2.60 as ALBI grade 1; −2.60< score ≤−1.39 as ALBI grade 2; and score >−1.39 as ALBI grade 3.

(3) The MELD score was calculated according to the formula: 3.8 × loge (bilirubin (mg/dl)) + 11.2 × loge (INR) + 9.6 × loge (creatinine (mg/dl)) + 6.4 × (etiology: 0 if cholestatic or alcoholic, 1 otherwise).

Table S1 Python packages or functions

Purposes	Packages	Version
Five-fold cross-validation	sklearn.model_selection.StratifiedKFold	0.23.1
AUC values	sklearn.metrics.auc	0.23.1
ROC plots	sklearn.metrics.plot_roc_curve	0.23.1
Diagnosis values	sklearn.metrics.confusion_matrix	0.23.1
HCR Features standardization	sklearn.preprocessing.StandardScaler	0.23.1
HCR Features selection	sklearn.feature_selection.VarianceThreshold;<br>sklearn.feature_selection.RFE	0.23.1
HCR Model finetune	sklearn.model_selection.GridSearchCV	0.23.1
HCR Modeling	sklearn.ensemble.RandomForestClassifier;<br>sklearn.linear_model.LogisticRegression	0.23.1
DL modeling	pytorch	1.10.1
Pretrained DL model	torchvision.models	0.11.2
DL data augmentation	albumentations	1.1.0
SHAP analysis	shap	0.40.0
Grad-CAM	captum	0.4.1

Note.—The setting of main parameters for random forest modeling is as follows: n_estimators = 200, max_depth = 2, max_features = 1
